# Concomittant pulmonary tuberculosis and borderline leprosy with type-II lepra reaction in single patient

**DOI:** 10.4103/0970-2113.59263

**Published:** 2010

**Authors:** Rajendra Prasad, Sanjay Kumar Verma, Rajni Singh, Giridhar Hosmane

**Affiliations:** *Department of Pulmonary Medicine, C.S.M. Medical University (erstwhile King George, Medical University), UP, Lucknow - 226 003, and G.S.V.M. Medical College, Kanpur, India*

**Keywords:** Leprosy, tuberculosis, coinfection

## Abstract

The concommitant occurrence of both tuberculosis and leprosy in a single individual are not an uncommon clinical condition but is being reported infrequently in literature. We report a case of leprosy, diagnosed previously and also diagnosed as pulmonary tuberculosis.

## INTRODUCTION

Leprosy is a chronic granulomatous disease principally affecting the skin and peripheral nervous system, caused by *Mycobacterium leprae*. It was discovered first time by Armauer Hansen in Norway in 1873. The infrequent occurrence of both tuberculosis and leprosy is based on the transmission dynamics of both infections.[1,2] The higher reproductive rate of *Tubercle bacilli* as compared to *Lepra bacilli* and degree of cross immunity within an individual do not allow both infections to occur simultaneously but there have been sporadic reports of co-existence of tuberculosis and leprosy in the same patients. Kumar B *et al*. reported that tuberculosis may occur through the spectrum of leprosy. We report a case of borderline lepromatous leprosy associated with type-II reaction and pulmonary tuberculosis in single individual.

## CASE REPORT

A 34-year-old, non- smoker male was admitted, in September 2007, to the Department of Pulmonary Medicine, as a follow through case of border line lepromatous leprosy. He had complaints of breathlessness, loss of appetite with cough with expectoration for last two and half months and skin lesions over face, forearm and dorsum of hands with exfoliative skin lesions over fore arm for last two months and recurrent haemoptysis for last 25 days. He was on medication, tab. Dapsone 100 mg daily, tab. Clofazimine 500 mg daily and cap, Rifampicin 600 mg, once a month, for the last nine months. He had also taken oral Prednisolone for more than three months duration about six months back, for neurological complications. There was no past history of tuberculosis as per information provided by the patient. Clinical examination revealed multiple hypo pigmented skin lesions varying in size from 2 to 4 cms over the trunk with thickened left ulnar nerve and nodular lesions over face, forearm and dorsum of hands [Figure 1a and 1b] and exfoliative skin lesions over fore arm [Figure 1c]. His resting pulse rate was 102/min and blood pressure was 112/74 mmHg and his respiratory rate was 26/min. His general examination revealed no significant abnormality. His respiratory system examination revealed coarse crepts localized to left infraclavicular and axillary areas. Initially he was put on symptomatic treatment for hemoptysis. Subsequently, his chest X-ray showed a large cavity with a mass filled opacity confined to left upper zone and fibrotic changes in right upper zone [Figure 2]. Computed tomography of thorax revealed fibro-consolidation with cavitation in the anterior segment of left upper lobe, fibrotic nodule in both anterior and posterior segments of right the upper lobe [Figure 3]. Thus a possibility of aspergilloma with other possibilities was raised. The clinical examination of the rest of the system revealed no abnormality.

**Figure 1:**
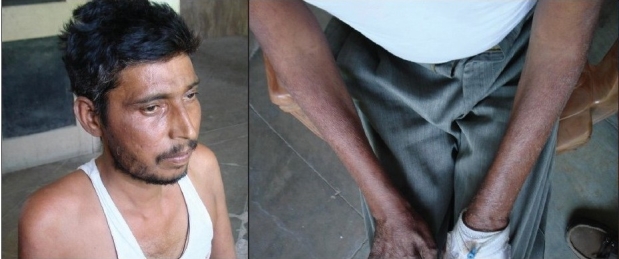
(a,b) Revealed skin lesions over face, forearm and dorsum of hands due to type-II lepra reaction

**Figure 1c:**
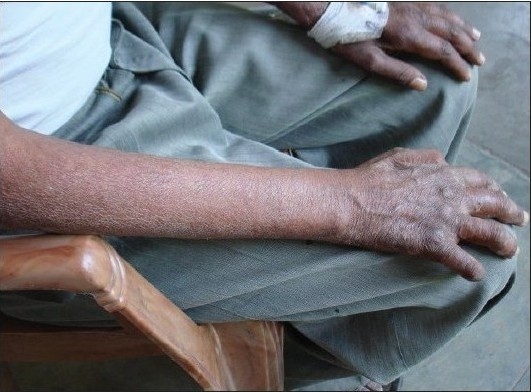
Revealed exfoliative skin lesions over forearm due to type-II lepra reaction

**Figure 2:**
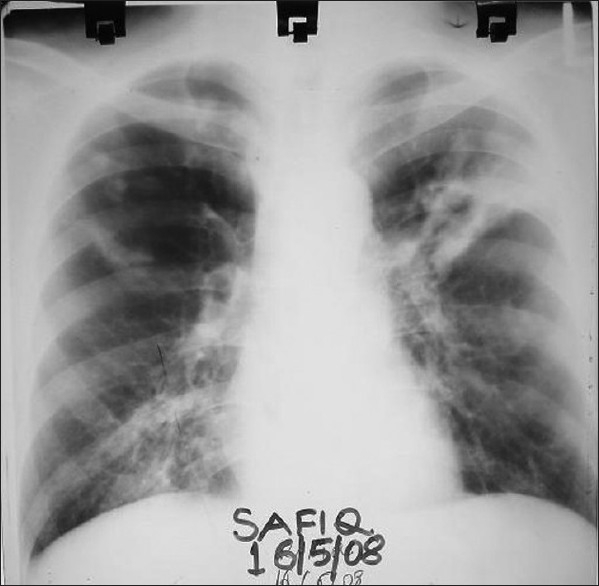
Chest X-ray reveals a large cavity with a mass filled opacity confined to left upper zone and fibrotic changes in right upper zone

**Figure 3:**
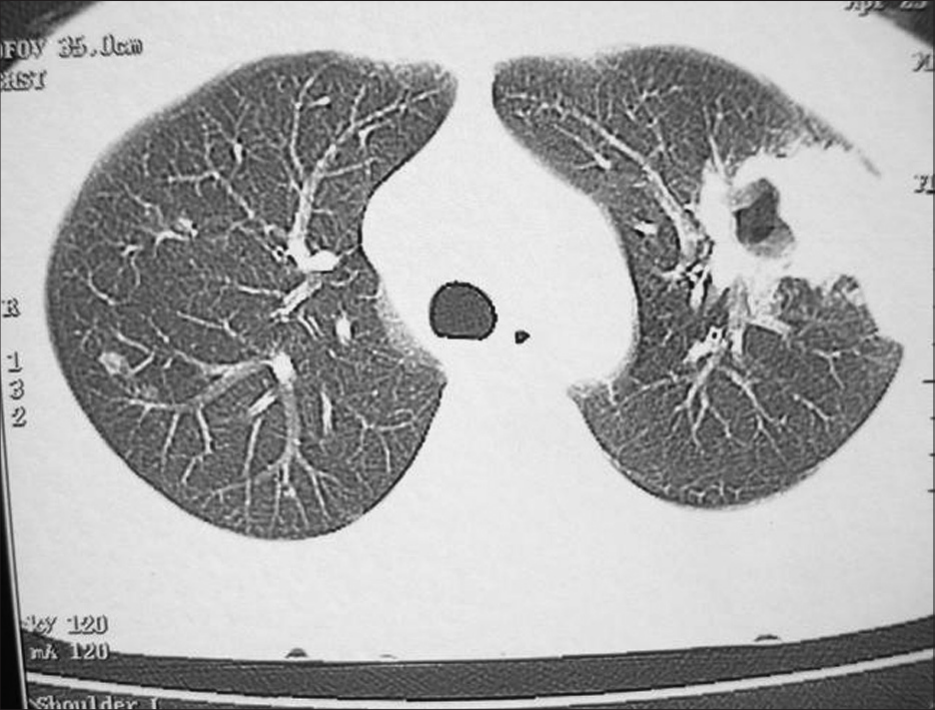
Computed tomography of thorax revealed fibro-consolidation with cavitation in anterior segment of left upper lobe with fibrotic nodule in anterior and posterior segment of right upper lobe

His blood biochemistry revealed total leucocyte count: 10,200/cmm, DLC: P 66 %, L 34 %. His PPD was 2219 mm induration. His sputum for AFB on three consecutive days was positive. Thus a diagnosis of pulmonary tuberculosis was confirmed. For leprosy, patient consulted the skin department, was diagnosed with borderline lepromatous leprosy (on the basis of slit skin smear) with type-II Lepra reaction (biopsy of nodular lesion revealed diffuse sheets of foamy macrophages centered around adenexal structures with heavy infiltration of lepra bacilli suggestive of Erythema Nodosum Leprosum). He was advised to stop Dapsone and started oral Prednisolone, Thaliodomide and Clofazimine.

Thus a final diagnosis of borderline leprosy with type-II lepra reaction with concomitant pulmonary tuberculosis was made. The patient was referred to DOTS clinic and started on category-I treatment. The oral prednisolone was subsequently tapered and stopped while the other antileprotic drugs continued. The patient's general condition improved and was on regular follow-up.

## DISCUSSION

Leprosy and tuberculosis continue to be prevalent in our country. Prevalence of tuberculosis is estimated to be 4.0 and 16.0 per thousand for bacteriologically and radiologically active tuberculosis cases respectively, while the national prevalence rate of leprosy in India is 0.88/10,000.[3] The great attention about leprosy and tuberculosis coinfection was carried out by Chaussinand in 1948, and concluded that the prevalence of leprosy was inversely related with the prevalence of tuberculosis. In some leprosy communities, however, tuberculosis appears[4] to be quite common. This may be the result of a small group of people unable to defend against either organism. Leprosy has failed to return in areas where tuberculosis has been controlled, but this may be because other conditions have changed (e.g., because of the introduction of bacille Calmette- Guerin).

The principal means of transmission of both leprosy and tuberculosis is by aerosol spread. The incubation period in leprosy varies from six months to 40 years or longer, while in case of tuberculosis it is only four weeks.

Review of literature suggest that the occurrence of leprosy and tuberculosis coinfection first time reported by Relvich AL *et al*. in 1954 and strongly argued that association of tuberculoid form of leprosy with tuberculosis was uncommon.[5] Gajwani[6] *et al*. 1968 and Gupta[7] *et al*. 1971, reported the association of tuberculoid type of leprosy with tuberculosis. This was further supported by Agnihotri MS *et al*, in 1974, who documented three cases of tuberculoid leprosy with tuberculosis[8] and Nigam P *et al*, (1979), who documented two cases of tuberculoid leprosy in association with tuberculosis.[9] On the other hand, most of the cases of tuberculosis were associated with lepromatous leprosy followed by borderline lepromatous leprosy, as observed in present case also.

The duration of gap between the development of leprosy and tuberculosis varied between two months to 10–15 years,[9,10] the study with largest data showed gap duration of about 10-15 years, where duration of tuberculosis in most of the cases was within six months (while in present case it was 11 months). Only two cases of tuberculosis were found to occur earlier than leprosy, [8,10] where as one study concluded that tuberculosis can occur during full spectrum of leprosy.[11]

It is well known that tuberculosis infection can develop with certain risk factors likes HIV infection, low socioeconomic status, silicosis, diabetes mellitus, gastrectomy, renal failure, organ transplant; these have been incorporated in the risk stratification in tuberculosis guideline[12] (no such type of risk factor present in present case). There are also other purporated risk factors such as smoking; rheumatic disorders and use of low dose immunosuppressive agents or glucocorticosteroids but substantive epidemiological data about these risk factors factor tuberculosis are scarce.[13] In case of leprosy, corticosteroids are used primarily in the treatment of type I (reversal) reactions and type II reactions and silent neuropathy. Chandrashekhar *et al*. (2000), reported development of pulmonary tuberculosis after corticosteroid intake in two cases of leprosy.[14] Agarwal *et al.* (2000), reported a case of leprosy and tuberculosis coinfection in a patient of renal transplant recipient and who had taken prednisolone, azathiorpine and cyclosporine for more than nine years[10] (while in the present case, he received corticosteroid for >3 months). In leprosy, majority of the cases reported were pulmonary tuberculosis, while in two cases of extra-pulmonary tuberculosis (tuberculosis of larynx[15] and cutaeneous tuberculosis[16]) were reported.

Diagnosis of leprosy was established in majority by slit skin smear but diagnosis by nasal smear and histopathological examination was also reported (while in present case by slit skin smear and biopsy also). Most common findings on chest radiographs were bilateral infiltrates[8,9,11,14,17] (as in the present case). Sputum smear for AFB was positive in majority of the cases with available data[8–10,14,17] (as in present case also).

Available data among three cases of leprosy with tuberculosis had lepra reaction including one of the author, in which one of them had type-II lepra reaction (ENL)[14] while one was type-I reversal reaction[18] (while in present case it was Type-II lepra reaction). Management of tuberculosis in leprosy coinfection does not change; with the same WHO treatment categorization i.e. Cat-I, Cat-II or Cat-III.

The detailed features of all the cases tuberculosis and leprosy coinfection and their comparison with present case are summarized in Table 1a and 1b.

**Table 1a:** Comparative analysis of leprosy-tuberculosis co-infection reported by various authors

Details	Authors						

	Gupta MC[6] (1971)	Vinik LA[19] (1971)	Agnihotri MS *et al*.[8] (1974)	Bhargava NC[20] (1976)	Nigam P *et al*.[9] (1979)	Gatner EM.[21] (1980)	Kumar B *et al.*[11] (1982)
No. of cases	NA	NA	3	NA	20	NA	9
Gap b/w dev. of leprosy and dev. of tuberculosis	NA	NA	NA	NA	10-15 years *Duration of tuberculosis in most of them was within 6 months	NA	NA
Types of leprosy	Tuberculoid leprosy	NA	Tuberculoid leprosy	NA	15 were of lepromatous, 3 of dimorphous and 2 of tuberculoid leprosy	NA	NA
Is tuberculosis developed first	NA	NA	Only in one case	NA	NO	NA	Tuberculosis was found to occur throughout leprosy spectrum
Is leprosy developed first	NA	NA	ND	NA	YES	NA	NA
Family history of tuberculosis	NA	NA	NO	NA	NA	NA	NA
Any risk factors likes use of corticosteroids/diabetes mellitus/ smoking/HIV/others were present	NA	NA	NO	NA	NA	NA	NA
TB: Pulmonary/extra-pulmonary	Pulmonary	NA	Pulmonary	Pulmonary	Pulmonary	NA	Radiological evidence of tuberculosis found
Descriptions lesions of tuberculosis on chest radiographs	NA	NA	B/L infiltrates	NA	Bilateral extensive pulmonary lessons were seen in 14 cases	NA	NA
Sputum for AFB	NA	NA	Positive in all of cases	NA	Positive in 80% of cases	NA	NA
Dx. of leprosy	NA	NA	Not seen	NA	NA	NA	NA
Lepra reaction*	NA	NA	Not seen	NA	NA	NA	NS

NA - Data not available; NS - Not seen

* Lepra type-I (reversal) or

* Lepra type-II (ENL)

**Table 1b:** Comparative analysis of leprosy-tuberculosis co-infection reported by various authors

Details	Authors						

	Flanagan PM *et al*.[16] (1993)	Agarwal DK *et al*.[10] (2000)	Srilakshmi MA *et al*.[17] (2003)	Lee HN *et al*.[18] (2003)	Chandrashekhar T. *et al*.[14] (2007)		Present author
No. of cases	NA	1	1	1	2		1
					Case-I	Case-II	
Gap b/w dev. of leprosy and dev. of tuberculosis	NA	2 M	10 Yr.	NA	3 M	2 Yr	11 M
Types of leprosy	LL	LL	LL	BL	BL	LL	BL
Is tuberculosis developed first	NA	YES	NO	NA	NO	NO	NO
Is leprosy developed first	NA	NO	YES	NA	Yes	Yes	Yes
Family history of tuberculosis	NA	NO	NO	NA	NA	NA	NA
Any risk factors likes use of corticosteroids/diabetes mellitus/ smoking/HIV/others were present	NA	Renal transplant recipient and had taken prednisolone, azathiorpine and cyclosporin	NO	NA	Corticosteroids	Corticosteroids	Corticosteroids
TB: Pulmonary/extra-pulmonary	TB of larynx	Pulmonary	Pulmonary	NA	Pulmonary	Pulmonary	Pulmonary
Descriptions lesions of tuberculosis on chest radiographs	NA	NA	B/L infiltrates	NA	B/L infiltrates	B/L infiltrates	B/L infiltrates
Sputum for AFB	NA	+ve and culture +ve	+ve	NA	+ve	+ve	+ve
Dx. of leprosy	NS	Biopsy	Slit skin smear				Slit skin smear
Lepra reaction*	NA	Seen	NO	Type -I	No	Type - II	Type -II

M - Month; Yr. - Year; NA - Data not available; NS - Not seen

* Lepra type-I (reversal) or *Lepra type-II (ENL)

## CONCLUSION

It is very important to screen out the clinical features of tuberculosis, in each and every patient of leprosy to avoid single drug therapy (e.g. Rifampicin, which is a highly bactericidal first line anti-tubercular drug), which may contribute to development of acquired drug resistance and reduced effectiveness of anti- TB treatment.
